# Autumn COVID-19 surge dates in Europe correlated to latitudes, not to temperature-humidity, pointing to vitamin D as contributing factor

**DOI:** 10.1038/s41598-021-81419-w

**Published:** 2021-01-21

**Authors:** Stephan Walrand

**Affiliations:** Cliniques Universitaires Saint-Luc, 1200 Brussels, Belgium

**Keywords:** Viral infection, Health policy

## Abstract

To determine the factor triggering the sudden surge of daily new COVID-19 cases arising in most European countries during the autumn of 2020. The dates of the surge were determined using a fitting of the two last months of reported daily new cases in 18 European countries with latitude ranging from 39° to 62°. The study proves no correlation between the country surge date and the 2 weeks preceding temperature or humidity but shows an impressive linear correlation with latitude. The country surge date corresponds to the time when its sun UV daily dose drops below ≈ 34% of that of 0° latitude. Introducing reported seasonal blood 25-hydroxyvitamin D (25(OH)D) concentration variation into the reported link between acute respiratory tract infection risk and 25(OH)D concentration quantitatively explains the surge dynamics. Several studies have already substantiated a 25(OH)D concentration impact on COVID-19 severity. However, by comparing different patient populations, discriminating whether a low 25(OH)D concentration is a real factor underlying COVID-19 severity or only a marker of another weakness that is the primary severity factor can be challenging. The date of the surge is an intrapopulation observation and has the benefit of being triggered only by a parameter globally affecting the population, i.e. decreases in the sun UV daily dose. The results indicate that a low 25(OH)D concentration is a contributing factor to COVID-19 severity, which, combined with previous studies, provides a convincing set of evidence.

## Introduction

**Figure 1:**
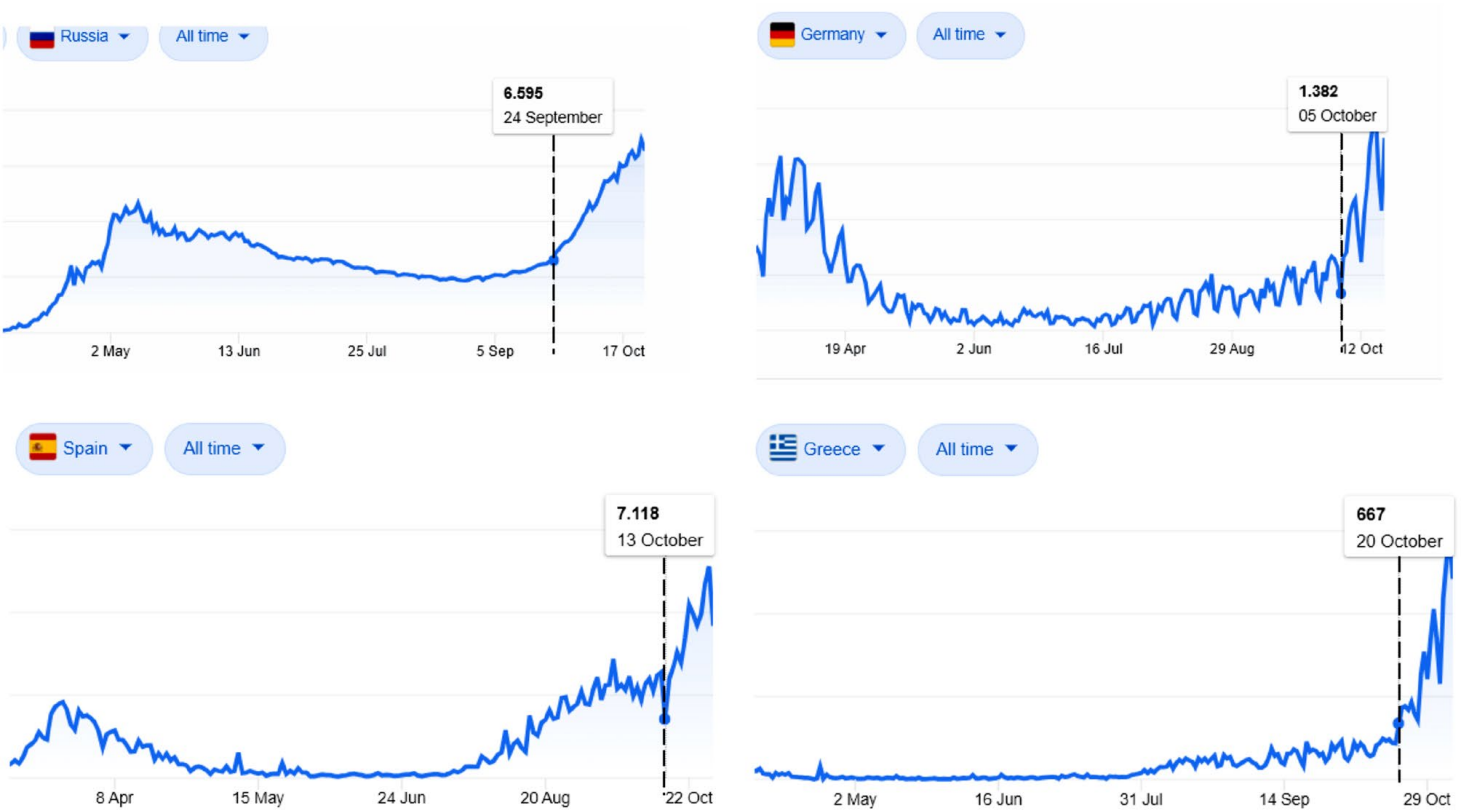
Typical examples of daily new COVID-19 cases (extracted from the statistics panel of the Google home page when searching “COVID” at www.google.com, last accessed 8th November 2020). All curves exhibit a clear surge in the growth rates.

Most European countries underwent an unexpected surge of daily new COVID-19 cases in autumn (Fig. 1), imposing new confinement rules and emergency lockdowns.

A commonly reported explanation is the decreasing temperature. The aim of this study is to challenge this assumption against a pure latitude impact.

## Materials and methods

### Data sources

The countries’ daily new COVID-19 cases, more exactly, the daily new SARS-CoV-2 seropositive cases*,* were obtained from the European Union agency European Centre for Disease Prevention and Control (https://www.ecdc.europa.eu/en/publications-data/download-todays-data-geographic-distribution-covid-19-cases-worldwide).

The country population weighted centre (PWC) latitudes were obtained from the Baylor University population resource (http://cs.ecs.baylor.edu/~hamerly/software/europe_population_weighted_centers.txt).

The averaged 2-week temperatures and humidity preceding the surge dates were computed from https://rp5.ru, which collects the archives of all airport weather stations in the world. For each country, an airport close to the PWC was chosen (see supplementary Excel file). The average temperature and humidity were computed between 8h00 to 20h00, outside this period, the population is mostly indoors.

School opening dates of 15 out of the 18 countries studied were found at https://eacea.ec.europa.eu/national-policies/eurydice/sites/eurydice/files/school_calendar_2020_21_0.pdf.

The theoretical sun UVB daily dose for vitamin D production, as a function of latitude and of the day of the year, was derived from the digitalization of Fig. 1B from reference^1^.

The reinforcement dates of the safety measure were obtained from the Public Health and Social Measures (PHSM) index of the WHO at https://who.maps.arcgis.com/apps/opsdashboard/index.html#/ead3c6475654481ca51c248d52ab9c61.

All these data are in the supplementary Excel file.

### Surge date determination

The date of the surge was automatically determined by fitting the two last months of the daily new COVID-19 cases with the empirical model:

1 $${N}_{c} \hspace{1mm}{e}^{\left({\alpha }_{c}+\left({\beta }_{c}-{\alpha }_{c}\right) l\left(t-{t}_{c}\right) \right) t}$$

where $l$ is the logistic function:

2 $$l\left(t-{t}_{c}\right)= \frac{1}{1+{e}^{-\gamma \left(t-{t}_{c}\right)}}$$

${t}_{c}$ is the date when the exponential coefficient, coming from the initial value ${\alpha }_{c}$, crosses the value $\frac{{{\alpha }_{c}+ \beta }_{c}}{2}$ before tending towards the final value ${\beta }_{c}$ when $t\to \infty$. $\gamma$ is the steepness of this changing. The date of the surge was defined as the time when 10% of $\frac{{{\alpha }_{c}+ \beta }_{c}}{2}$ was added to ${\alpha }_{c}$; this choice corresponds to the date when Eq. (1) visually becomes different from the monoexponential (see supplementary file). $\gamma$ was assumed to be country independent, as we searched for an impact of latitude on its own. This further allows us to prevent overfitting of the data noise by a steepness tuned for each country.

Note that as the exponential coefficient varies with time, the doubling time around the surge date is not simply ln(2) divided by this coefficient.

### Dynamic models of new daily cases

To evaluate the impact of UV insolation on the new daily case dynamics, we consider the simple model:

3 $$\frac{dN(t)}{dt} = k\left(t\right) N(t)- \rho N(t)$$

where $N\left(t\right)$ is the total number of persons who have active SARS-CoV-2 at time *t*, its derivative is the new daily cases, and $k\left(t\right)$ is the mean effective contagiousness of an infected subject, which mainly depends on his coronavirus release in air and on materials, on the closeness and frequency of his contacts with other subjects; *ρ* is the recovery rate.

The solution of Eq. (3) is:

4 $$\frac{dN(t)}{dt} = {N}_{c} \left(k(t)-\rho \right) {e}^{{\int }_{0}^{t}\left(k({t}^{^{\prime}})-\rho \right) d{t}^{^{\prime}}}$$

where Nc is the initial number of infected persons in country c.

If the contagiousness is constant, the daily new cases follow a monoexponential increase or decrease:

5 $$\frac{dN(t)}{dt} = {N}_{c} \left(k-\rho \right) {e}^{\left(k-\rho \right) t}$$

We will consider two impacts of UV insolation: outdoor SARS-CoV-2 inactivation and blood 25-hydroxyvitamin D (25(OH)D) concentration.

### Impact of outdoor SARS-CoV-2 inactivation by solar UV

The active fraction survival of SARS-CoV-2 f. under a constant UV insolation R is governed by:

6 $$f\left(\tau \right)= {e}^{-\alpha R \tau }$$

where α is the solar UV sensitivity of SARS-CoV-2, and *τ* is the insolation duration. A recent detailed analysis^2^ shows that in Europe, *τ*_*90*_, i.e. the noon solar insolation duration needed to inactivate 90% of SARS-CoV-2, approximatively linearly increases between August and October from 60 to 150 min for southern countries and from 100 to 250 min for northern countries.

Considering this inactivation as the single effect varying *k(t)*, we obtain:

7 $${k}_{1}\left(t\right)= k(0)\hspace{1mm} {e}^{- \frac{\tau }{{\tau }_{90}(t)} ln(10)}$$

where $\tau$ is the mean time between the infection of a material and the contact with this material by a noninfected person.

**Figure 2:**
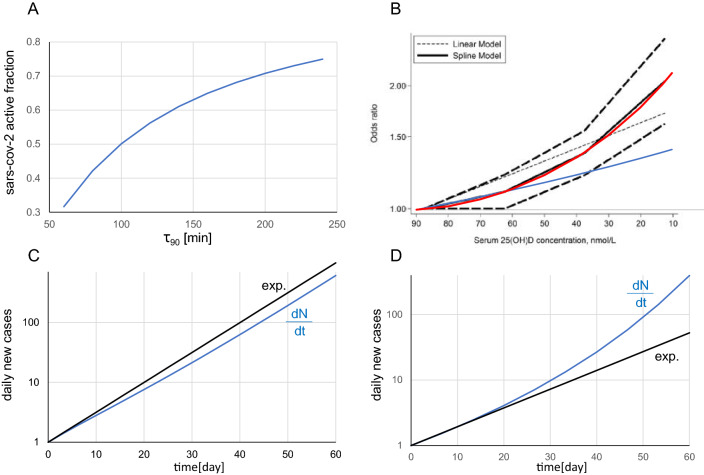
**(A)** Survival of the active SARS-CoV-2 fraction after 30 min of solar UV insolation as a function of τ_90_. **(B)** Solid black line: acute respiratory track infection (ARTI) risk during cold and influenza epidemics as a function of the 25(OH)D concentration (reprinted from^3^ according to the  CC BY 4.0 licence terms (https://creativecommons.org/licenses/by/4.0/); red and blue curves: power exponential (Eq. 8) and first point monoexponential fits added by the present author. In both graphs, the corresponding date runs from left to right. **(C,D)** Blue curve: corresponding new daily cases to **(A,B)** obtained by numerically integrating Eq. (4) (see excel file). In both integrations, k_0_ was fitted to obtain a typical threefold increase in the last 10 days (see Fig. 1), and ρ was neglected (surge phase). Black curve: mono-exponential curve.

Figure 2A shows a typical *k*_*1*_*(t)* curve for *τ*_*90*_ ranging from 60 to 250 min, and $\tau$ = 30 min ($\tau$ can be modified in the supplementary Excel file) and figure 2C its corresponding daily new cases (Eq. 4).

### Impact of blood (25(OH)D) concentration

A meta-analysis of 24 studies reporting the association between 25(OH)D concentration and the risk or severity of acute respiratory track infection (ARTI) during cold or influenza epidemics^3^ shows that the risk follows a power-exponential relation (Fig. 2B).

Assuming that COVID-19 risk similarly depends on 25(OH)D concentration and considering this risk increase as the single effect varying *k(t)*, we obtain

8 $${k}_{2}\left(D(t)\right) = k(D(0))\hspace{1mm} {e}^{0.00069{ \left(90-D(t)\right)}^{1.6}}$$

where *D(t)* is the 25(OH)D concentration expressed in nmol/L units at time *t*.

Figure 2D shows the corresponding daily new case (Eq. 4).

Studies in Europe^4–8^ reported a seasonal 25(OH)D concentration drop of 20–26% between August and October (Table 1). Two longitudinal studies^6,7^ followed one single cohort over 12 months, with one^7^ reporting the 25(OH)D concentration curves for each subject allowing a rough estimation of the intraindividual standard deviation, giving a drop of 26 ± 25% in normal subjects (age 31 ± 3 years). This standard deviation could still be higher in the country population with the presence of older and chronically ill subjects. As a result, more than 15% of the European active population could suffer from a 25(OH)D concentration decrease larger than 50% from August to October. Assuming that the COVID-19 risk follows Fig. 2B and that the whole population has an initial D = 90 nmol/L value, a rough estimation of the k(t) surge can be computed (see Excel sheet “vitD severity” S-AA for the numerical integration result) as:

**Table 1:** Monthly seasonal 25(OH)D concentration studies.

Country (ref)	PWC lat. (deg)	Population	Type	n ± std per month	Age (year)	Drop ± std (%)
Sweden^4^	59.0	Blood donors	≠ cohorts	45 ± 9	41 ± 13	22
Denmark^5^	55.9	Blood donors	≠ cohorts	16 ± ?	24–89	23
UK^6^	52.8	1958 birth	Longitudinal	6789	45 ± 0	26 ± ?
Netherland^7^	52.1	Nurses	Longitudinal	8	31 ± 3	26 ± 25
Poland^8^	51.7	Athletes	≠ cohorts	229 ± 55	25 ± 1	23

Right column: 25(OH)D concentration drop between August and November.

9 $$k\left(Oct\right)= \frac{1}{\sqrt{2\pi } 23} {\int }_{0}^{\infty }k\left(D\right) {e}^{- \frac{{\left(D-67\right)}^{2}}{2 \times {23}^{2} }} dD \hspace{2mm}k\left(Aug\right)=1.18 \hspace{2mm}k(Aug)$$

In the integral, $\underset{D\to 0}{\mathrm{lim}}k\left(D\right)= \infty$ but due to the higher exponential power of the Gaussian distribution, i.e. 2, versus that of *k(D)*, i.e. 1.6, the integral does not diverge and can even be truncated at *D* = *0*. The integration was extended above the initial D = 90 nmol/L value because one patient in the cohort^7^ exhibited a concentration increase rather than a drop.

## Results

Table 2 shows the fitting results (all data and fitting processes are provided in the supplementary xlsx file). For Sweden, the new daily cases were constant before August, preventing the computation of the β/α ratio. Although that the parameters are accurately measured, they suffer from the limitations that, besides the latitude, they are not population weighted.

**Table 2:** Temp, hum: mean country temperature and humidity during the 2 weeks preceding its COVID-19 surge date.

Country		School opening	Temp (°C)	Hum (%)	PWC lat (deg)	Surge start	Day	Solar UV (%)	β/α
Greece			23	52	38.7	Oct 21	295	37	1.21
Portugal		Sept 14	18	79	39.7	Oct 10	284	41	1.06
Spain			18	47	39.8	Oct 14	289	39	1.37
Bulgaria		Sept 14	22	55	42.8	Oct 06	281	39	1.16
Italy		Sept 14	20	70	42.9	Oct 06	281	39	1.02
Serbia		Sept 01	15	72	43.8	Oct 18	293	32	1.13
Croatia		Sept 01	17	68	45.3	Oct 10	284	34	1.06
Slovenia		Sept 01	13	83	46.2	Oct 12	286	32	1.17
Switzerland		Aug 17	14	78	47.0	Oct 04	279	34	1.25
France		Sept 01	14	80	47.2	Oct 11	286	31	1.09
Austria		Sept 08	13	71	47.8	Oct 16	290	28	1.10
Belgium		Sept 01	21	53	50.8	Sept 25	270	34	1.02
Germany		Aug 14	14	73	50.9	Oct 06	281	29	1.15
Netherland		Sept 01	18	66	52.1	Sept 26	271	32	1.08
UK		Sept 01	18	67	52.8	Sept 19	263	35	1.14
Russia			18	59	54.3	Sept 13	258	37	1.06
Sweden		Sept 01	15	72	59.0	Sept 13	258	30	
Finland		Aug 14	15	73	61.8	Sept 10	254	27	1.11

*PWC lat* latitude of the country PWC, *Sun UV* theoretical sun UV daily dose at the surge date, expressed as a fraction at the PWC latitude versus the latitude 0°.

Table 3 shows the reinforcement implemented during September and October of the existing safety measures (see country pages in Excel file to see the intensity level of each safety measure). With regard to the delay between infection and contagiousness of a subject, only two reinforcements (in bold) could have delayed the surge date. The abundance of measured reinforcements within the two weeks following the surge date provides evidence of the surge threat.

**Table 3:** Safety measure reinforcement dates in the September–October period.

Country		Surge start	Mask	Schools	Work	Gathering	Movement	Int. travel
Greece		Oct 21					Oct 16	
Portugal		Oct 10	Oct 22				Oct 22	
Spain		Oct 14		Oct 14			**Oct 02**	
Bulgaria		Oct 06		Oct 27	Oct 06	Oct 06	Oct 04	
Italy		Oct 06	Oct 05	Oct 13			Oct 22	
Serbia		Oct 18						
Croatia		Oct 10				Oct 27		
Slovenia		Oct 12		Oct 20	Oct 20	Oct 17	Oct 17	
Switzerland		Oct 04	Oct 25	Oct 22	Oct 22	Oct 22		
France		Oct 11			**Sept 10**	Oct 27	Oct 27	Oct 27
Austria		Oct 16		Oct 15	Oct 15		Oct 15	Oct 15
Belgium		Sept 25			Oct 05	Oct 15	Oct 15	
Germany		Oct 06		Oct 20	Oct 20	Oct 12	Oct 20	
Netherland		Sept 26			Oct 15	Sept 29	Oct 14	
UK		Sept 19		Oct 20	Oct 07	Sept 12	Sept 20	
Russia		Sept 13	Oct 15	Oct 02			Oct 05	Oct 11*
Sweden		Sept 13			Oct 27			
Finland		Sept 10	Sept 30	Oct 22		Oct 12		

Bold: reinforcement could have reduced the surge.

*Decrease of the safety measure.

**Figure 3:**
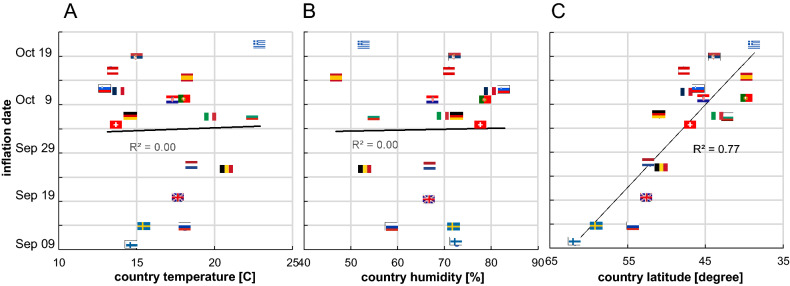
COVID-19 surge date as a function of country mean temperature **(A)** and humidity **(B)** during the 2 preceding weeks and as a function of country PWC latitude **(C)**, pointing to vitamin D as one of the primary factors (flags link countries between graphs).

Figure 3A,B clearly prove no correlation with temperature or humidity, while Fig. 3C clearly shows an impact of country latitude.

**Figure 4:**
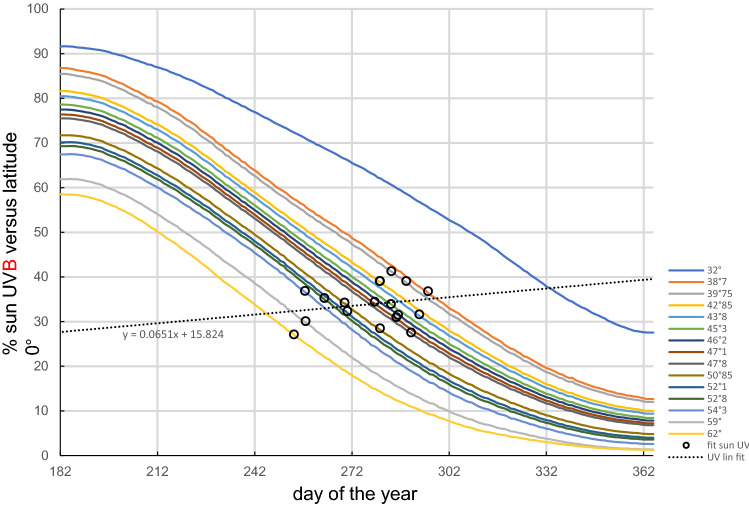
Solid curves: theoretical sun UVB daily dose for vitamin D skin production under a clear sky corresponding to the 18 PWC country latitudes plus the 31° latitude (derived from Fig. 1B in^1^). Black circles: country surge dates positioned on their corresponding latitude curve.

Figure 4 clearly shows that the surge dates set on the sun UVB daily dose as a function of latitude demonstrate an impact of the sun UVB daily dose.

**Figure 5:**
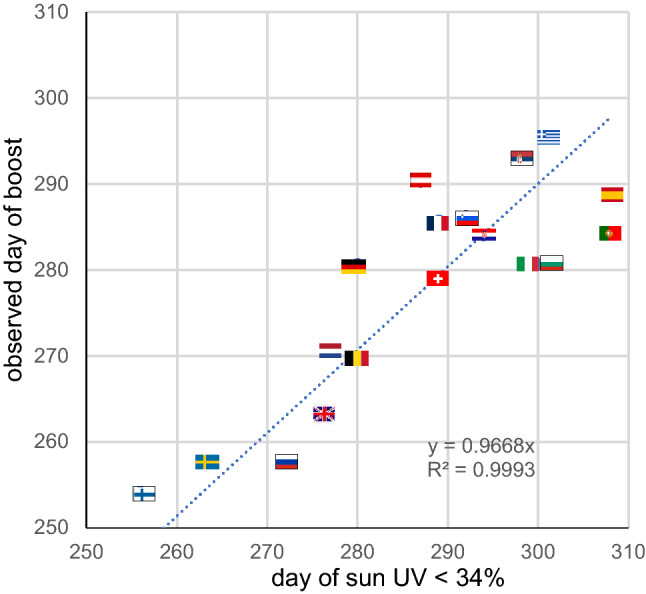
Observed day of the second COVID-19 wave surge as a function of the day when the country sun UVB daily dose drops lower than 34% of that at latitude 0°. Trendline forced to intercept (0,0).

Figure 5 shows that the day of the second wave surge is predicted well by the time when the sun UVB daily dose of the country becomes lower than 30% of that at latitude 0°.

## Discussion

Many studies support an impact of low 25(OH)D concentrations on respiratory impairment in coronavirus or viral diseases^9^ and recently on the COVID-19 pandemic as well (see^10^ for a detailed review and analysis of 14 studies reporting such impacts). Low 25(OH)D concentrations are also more prevalent in populations at risk, i.e. aged people^11,12^, obese patients^13^, people with coloured skin living in high latitude countries^14^ and diabetic patients^15^.

However, by comparing COVID-19 severity between different populations, determining whether the 25(OH)D concentration is a real factor of COVID-19 severity or only a marker of another weakness that is the primary severity factor can be challenging.

The date of the surge is an intrapopulation observation and has the benefit of being triggered only by a parameter globally affecting this population. There is no correlation with temperature, humidity, or school opening dates (see Excel file), but there is an impressive latitude correlation (Fig. 3). The remaining common parameter affecting these populations monotonically at different times depending on latitude is the sun UV daily dose (Fig. 4).

This UV index dependence was already observed for influenza epidemics^16^, although the temperature dependence appeared more important. A global seasonality study also evidenced a monthly correlation between other pre-existing human seasonal coronavirus activities and temperature and humidity^17^. However, this study did not consider latitude as a confounding factor, and on a monthly scale, there is a correlation between temperature-humidity and latitude. On the daily scale used in the present study, this correlation no longer exists as each country is temporally affected by different wind directions. This feature allows us to clearly discriminate between temperature-humidity and latitude impacts.

Decreasing sun UV insolation can impact COVID-19 dynamics in two ways: by decreasing outdoor SARS-CoV-2 inactivation or by decreasing the population 25(OH)D concentration.

However, many European countries were able to break the surge in November by implementing additional safety rules. Activities where people cannot wear face masks, such as collective sports or relaxation in pubs and restaurants, were forbidden, and festive activities where people often forget distancing recommendations were forbidden. In contrast, the population continued their professional and outdoor relaxing activities wearing face masks at work, in public transport, in itinerant outdoor markets (European use) and in parks. The success of these rules supports the major role of airborne transport of SARS-CoV-2 versus contamination by outdoor contact with infected material.

Equations (7, 8) clearly illustrate that the potential impacts on COVID-19 dynamics of outdoor SARS-CoV-2 inactivation and a decrease in 25(OH)D concentration are fundamentally different. Indeed, Fig. 2B indicates that even if the 25(OH)D concentration slowly decreased after the summer solstice, its impact on contagiousness becomes increasingly important with time and leads the dynamics to strongly diverge from a monoexponential trend after a while as shown in Fig. 2D, which is in line with the data in October. In contrast, Fig. 2A indicates that the impact of outdoor SARS-CoV-2 inactivation decrease on COVID-19 contagiousness becomes increasingly less important with time, which should correspond to an increase in July–September, moving towards a doubling time stabilisation, as shown in Fig. 2C.

Another feature discards the potential role of solar UV inactivation: in Europe, people spend the majority of their time indoors, so even if contact with a contaminated surface can be a source of transmission, the contact probability is lower outdoors than indoors where solar UV inactivation is absent. This is in line with a one week recent study showing that outdoor contamination is much less frequent than indoor ones^18^.

The obtained β/α ratios range from 1.02 to 1.37 (Table 2), which is in line with the estimated ratio of 1.18 as Eq. (9) neglects any country dependence and was based on a small cohort (n = 8) follow-up.

The positive linear slope of the sun UVB threshold versus the country latitude (Fig. 4) is also in line with the fact that, due to natural adaptation, populations have increasingly pigmented skin when the latitude decreases. As a result, skin vitamin D production in northern populations is affected by the sun UVB decrease in a slower manner than that of the southern populations. Figure 4 is also in line with the low population mortality observed within ± 35° latitudes^19^ and reported in Hubei located at 31° latitude, because these regions are above the sun UV daily dose 34% average threshold most of the year.

The present study thus suggests that a low 25(OH)D concentration is a contributing factor of COVID-19 severity, as already shown by previous studies^10^, which together constitute a convincing bundle of evidence. By increasing the coronavirus load in the respiratory tract, the contagiousness in the population is also increased, starting a chain reaction that explains the wave surge.

This study has three strengths. The utilisation of the date of the surge is not dependent on the differences between the safety measures implemented in the countries but can only depend on the change in a global parameter affecting the whole country population. The correlation analysis on a daily scale prevents the interpretation from being blurred by the seasonal latitude-climate correlation existing on a monthly scale. Using the relation reported between ARTI and 25(OH)D concentration together with the reported seasonal 25(OH)D concentration variation, the derived prediction of the daily new case slope increase is in line with the observation.

The study has several weakness. Some countries have a homogeneous population distributed at a few latitudes, such as France, Germany, and Russia, which could twist or blur the correlation. Access to reported regional new daily cases should be very valuable. The surge intensity analysis was performed using the 25(OH)D concentration reported in a small volunteer cohort. Observed autumn 25(OH)D concentration decrease for several countries should be helpful to further increase the confidence in the vitamin D status contribution.

## Conclusion

As already evidenced by previous correlation studies^10^, a low 25(OH)D concentration should be considered a contributing factor to COVID-19 severity.

Europe and the northern USA are starting a long COVID-19 crisis this autumn, as they will return to a level above the October sun UV daily dose only at the end of March 2021.

Measures to reduce the pandemic severity during the coming winter using controlled preventive vitamin D supplementation should be considered^10,20^.

## Supplementary Information

Supplementary Information 1.

Supplementary Information 2.
